# American Medical Association, Section on Stomatology

**Published:** 1902-09

**Authors:** 


### AMERICAN MEDICAL ASSOCIATION, SECTION ON STOMATOLOGY.

*First Session, Tuesday, June 10, 1902.*

THE Section was called to order by the Chairman, Dr. A. H. Peck, of Chicago.

Upon motion of the secretary, Dr. Eugene S. Talbot, of Chicago, the programme was accepted as published.

Dr. R. R. Andrews, of Cambridge, Mass., was called to the chair and Dr. Peck delivered the Chairman's Address, entitled "Physical Diagnosis as related to Dental College Curricula."

(For Dr. Peck's Address, see page 565.)

*Dr. Peck.*—It has been said that those in doubt as to a subject should refrain from writing at all. I have had some difficulty in selecting a subject on which to write for this occasion, and I am not sure but that you will come to the conclusion ere I have finished that I should have refrained from writing. I thought of writing along the line of the history of this Section, but that is printed in the Journal of the Association, and therefore was not advisable. A history of the work of the Section in the profession did not seem a proper subject, so I have gone out of the usual line of addresses of chairmen of such bodies as this, and have prepared a paper upon a specific subject,—"Physical Diagnosis as related to Dental College Curricula."

### DISCUSSION.

*Dr. J. W. Williams,* Boston.—There is a matter of great importance,—knowing how to refrain from too long continued operations. Some time ago I read a paper at New Orleans upon the importance of rest with the production and continuation of strength. It is a matter which I have studied for years, and find it of vital importance. In all vital forces there is an intermission. The heart has its times of rest; also the breathing. I have demonstrated the difference between the intermittent action of the muscles and continuous action by having a boy hold out his arm for half an hour. The muscles would soon become exhausted, but with the momentary intermissions in work, the muscles are strengthened. It has been too much disregarded that we must have rest at proper intervals.

There is also the importance of physical diagnosis, to determine whether a patient can bear not only continued operations on the mouth, but certain operations which may be short, as when a number of teeth are taken out at a time. My old preceptor would not take more than two or three teeth out at once. Even though the patient is anæsthetized, there is a certain shock to the vital forces greater than from the loss of fingers, because the operation is done in the mouth, the "gate of life," as it is called. The skin being more highly organized, there is more vital effort required in its healing than in a flesh wound. There are several recorded cases of great and serious depression of the constitutional vitality following the extraction of large numbers of teeth. In one case, in which ether was given, the patient after the removal of fourteen teeth was confined to bed from exhaustion not only from the shock, but from the effort of the system to heal the wounded surface, and died in about a fortnight. In another the patient lingered for about three weeks. Such occurrences seem to be entirely unknown in general practice. I have known instances of vital prostration of several years' duration from the extraction of a large number of teeth. The removal of the teeth from the alveoli seems to have a far more depressing effect than the smoothing down of the teeth for the preparation of a plate fitted over them. I have seen several old persons whose lives, I think, were prolonged by having the roots remain and their mouths put in perfect condition in this way.

I wish that the matter of refraining from prolonged and serious operations either lying or sitting might be so continuously alluded to and condemned that it would be more generally considered reprehensible to do such operations. It is a vital principle of continued health and strength that there be intermission of rest, with a quiet effort at proper intervals.

*Dr. Vida A. Latham,* Chicago.—I think a subject which should precede physical diagnosis is physiology. There I believe to be the greatest mistake of all our dental schools. In fact, I may say I regard it as a disgrace that a country like America should give no course of physiology in the dental colleges. The student may know nothing about the relations and functions of the body, but in nine cases out of ten he is an excellent anatomist. In the study of anatomy there are a certain number of operations and dissections, and a man, therefore, pays attention to that kind of work, whether he is a young medical man or a young dentist. Personally,

I am much in favor of urging the dental profession to have a better course in physiology, especially of a practical laboratory nature. Let a student take a heart, let him examine it, see it beating in the animal, and let him try the experiments and see the effects of the drugs. In that way only can we teach our physiological work. As a teacher of pathology I have had that stumbling-block to encounter to such a degree that I believe the average dentist cannot properly teach because there is no distinct training in practical physiology.

I would ask that the courtesies of the floor be extended to Professor Gage, of Cornell, with whose work as a teacher of histology and physiology we are all familiar.

*Professor Gage,* Cornell.—I have just had a year of teaching, and I would like to learn something in readiness for next year. It seems to me that the doctrine of Dr. Peck's paper is perfectly sound, and I am exceedingly glad to hear Dr. Williams speak of the undesirability of long-continued operations with the demand upon the vital force. As Dr. Latham says, if the student has a thorough training in physiology he will better appreciate what he can do with the living organism than in any other way. Naturally, to thoroughly appreciate physiology, he must have a knowledge of anatomy. I would welcome every effort to increase the knowledge of the members of the dental profession, and physiology I would say is a fundamental subject for the student to learn. Anatomy, of course, is necessary, but physiology is really at the base of all processes that make dentistry valuable. The man who says that the medicine or operation cured his patient, I know, is not a physiologist; but if he says he gave nature a chance, I know he is a physiologist.

*Dr. G. V. I. Brown,* Milwaukee.—The paper of the chairman, it seems to me, calls for more than an ordinary discussion. What Dr. Latham said about the course in physiology is true. We have not such a course as we would like to have as a foundation, yet with such foundation as we have we must do the best we can. In the college in which I have, until recently, had charge, we have had to cover the subject of physical diagnosis in a practical way. I have found several difficulties, one of which was to convince others with whom I had to associate of the end and aim of this course. In the second place, the student himself has much more to say than is ordinarily taken into account, and the students as a body object to having additions to the curriculum. Physical diagnosis is considered a dry thing unless the lecturer is particularly fortunate in his presentation. To make the study interesting we conceived the plan of dividing the Senior class, have the men strip to the waist, and have the other half of the class study, under the director, auscultation and the other simpler means of physical diagnosis. This we found very successful, and we have arranged to add to the plan this year by taking the men to the county hospital, where the might see, in addition to the comparatively perfect specimens in their class, the pathological conditions in hospital patients. This could be done in almost any college. It is an entering wedge, and, having instilled the idea of the necessity of such a course, we may in time be able to secure the teaching desired. I think it would be wise to develop the course in some such practical way. As Dr. Latham has said, we ought to have the foundation stone of a complete course in physiology.

*Dr. G. F. Eames,* Boston.—I have been much interested in the matter of widening the field which the dental profession shall cover. I devoted some years to the study of the throat and nose, and I am sure that those studies have assisted me in dental practice. I have devoted other years to the study of the general practice of medicine, and I am sure that also has assisted me; but I have been negligent in my study, and I have felt unable in some cases to make an accurate diagnosis in regard to conditions of the heart and lungs when it was necessary to give the character of the pathological condition. I have felt in later years that the physician who is doing such work daily should make diagnoses for me. I feel that we are just in the beginning of this matter suggested by the chairman. It is a most excellent idea, and one which will raise us to a much higher level than we have ever before attained. We must give the matter more study and put it into actual practice, in order to cultivate the ear in auscultation and the hand in percussion and palpation. In that way only can the dentist become proficient in the diagnosis of pathological conditions. I feel that by the experienced man much for dental purposes can be told by simply looking into the face of the patient. If he is then able to make an accurate diagnosis, let him do it; if not, the case should be given into the hands of a man who can.

*Dr. G. Lenox Curtis,* New York.—It seems to me that, to give the dental practitioner what is needed in the line of physiology, the dental schools should be connected with medical institutions, where they might receive their instruction in all the essential medical branches, the same as does the medical student. It is essential that dentists should have a knowledge of physiology as well as of anatomy, and, as some others have said, they lack perhaps in this more than in any other department. I think the matter is highly important, and I should like to see some action taken at this meeting recommending, or, perhaps, making it stronger, appointing a committee to see if such a thing could not be brought about in dental schools.

*Dr. M. L. Rhein,* New York.—This is a subject in regard to which I believe we are all in accord with the essayist. The only point of criticism, if it might be termed such, that I would make is that the paper did not go far enough. Education in this specialty of medicine should be as far-reaching as in any other. To physical diagnosis I would add a study of the urine and the blood. I coincide with the severe criticism of Dr. Latham upon the lack of thorough fundamental education in dental institutions. I believe the solution of the problem lies in the association of the dental institution with the medical in the universities. I think that, so far as possible, this Section should teach that doctrine. It is folly for us to attempt to improve the form of dental education at present in the United States, for the simple reason that the majority of members of the National Association of Dental Faculties, who control the system of education of dentistry in this country, are owners of preparatory schools, and their interests have never tended towards the higher education of dentists.

*Dr. Eugene S. Talbot,* Chicago.—I consider the paper of our chairman very timely. There are a great many changes which should take place in medical as well as dental education, and I second the remarks of Dr. Rhein in regard to this matter. The time has come when we must take some stand in regard to dental education. Yesterday the National Board of Medical Faculties spent some hours in talking over the relation of dental colleges to medical colleges, and it was only by a mere chance that the matter was laid over until next year, and that they did not divorce the dental schools entirely from the medical. If there is one class of patients to whom a knowledge of the general condition of the body is necessary more than another, it is the dental patients, and the dentist knows little or nothing about the conditions under which these patients come to him for operation. The nervous system, upon which we as dental students and practitioners have been so poorly educated, has more to do with our patient than any other one thing. The development of the face, jaws, and teeth, the decay of the teeth, and diseases of the alveolar processes are all based upon trophic neuroses, yet we know nothing about the nervous system in that respect. Such patients are neurotics, and, as Dr. Rhein says, we should be educated thoroughly as medical practitioners in order to treat our patients successfully. This Section should insist that dental students shall be educated in the fundamental principles of medicine with medical students. It has been my privilege this winter to lecture in five or six of our university dental schools, and I am surprised to see the price that the dental faculties are paying for the sake of isolating these two medical departments. We have in the West, and I suppose you have in the East, three or four different departments, with different sets of teachers, with expensive buildings and apparatus for the teaching of the different departments of medicine, when all might be educated in one building and with one set of teachers. I think the time will come very soon when this subject will be taken up from an economic as well as an educational stand-point. I am very glad that our chairman has presented the subject to us in the way he has.

*Dr. D. E. Hoag,* Brooklyn, N. Y.—I voice the sentiments already given by some of the members present. I was not fortunate enough to hear the paper, but the advancement of dental education along the lines suggested is not so slow as we think. Although dentistry is old in mechanical science, it has only been a few years since it was put upon a professional basis. On the other hand, there has been a lack of union, a lack of sympathy, between the dental and medical professions. The dental profession will soon be on a higher plane, because better men are entering it, and the requirements are also becoming higher.

The remarks made relative to physical diagnosis were very pertinent, but, from my point of view, I regard physical diagnosis as an art. The study of physiology, histology, and kindred subjects is more distinctly a science. Though we study an art, we can only become proficient by practice. A man may take a course in physical diagnosis, but unless he is in a position to practise it, he becomes very rusty. A dentist does not always have the needed opportunity.

The future will show a closer union betwixt medical and dental schools. I began the study of dentistry with the sole idea of studying it and nothing else. During my first year in college I saw how much there was in medicine, so that I took the other course in addition. I was very fortunate in having for one of my teachers Dr. Latham, who is here to-day. She imbued me with a sense of diligence in those branches which men in college regard as almost wholly useless.

*Dr. E. A. Bogue,* New York.—The idea which Dr. Peck has presented to us I think none will venture to oppose, but what the ordinary dental student is able to digest and put into practice is a very different thing. It seems to me, however, that we are moving towards a oneness with the vast medical world with considerable rapidity; not because of our efforts, perhaps, but because of what we may term the forces of development. Society around us is becoming more intelligent, and is demanding more of a dentist than formerly. When I was a child a man who was a good repairer of watches was therefore expected to be a good dentist, because his fingers could do good mechanical work. Whether we are able to superadd physical diagnosis and a thorough knowledge of physiology to the present curriculum is a question which those more engaged in teaching than I are better qualified to answer. The curriculum as it stands to-day is nearer what the entire medical curriculum was twenty-five years ago than we are wont to imagine.

As to the extension of the dental curriculum, I hope it will be carefully considered whether the student to-day admitted to the dental college is able to carry additional work.

*Dr. J. L. Williams,* Boston.—It is not so much what a student likes to study, but what he ought to study. If the instruction is deficient, it should be made adequate. This matter of resolution I think is a good suggestion, and I think it is the natural outworking of forces. There is among the laity a more general appreciation of the idea that the human system is sympathetic in parts and controlled by similar principles in health and disease. Students have often said to me that they would like to take a medical course in connection with the dental, but that they have not the time. I tell them that they are right, and that the specialty of dentistry, as any other specialty, belongs to the human system.

*Dr. R. R. Andrews,* Cambridge, Mass.—I believe thoroughly in having a medical education in connection with the dental. I put my own boy into school with the idea of having him secure a medical education and the dental afterwards. He, however, chose surgery. I heartily endorse the paper, and I think cases come to us needing just such examinations as suggested by the chairman. The general trend of dental education in the East is upward. We have added another year, making the course four years in one of our Eastern colleges. This will soon be adopted, I think, by Harvard, and this will soon be followed, I believe, by the requirement of an entrance examination equal to that in medical schools.

*Dr. Peck* (closing the discussion).—I will take time only to say that I am very much gratified with the uniform approbation with which the subject presented in the paper has been accepted by you. This is a subject that has engaged my attention for some time. I have been thoroughly imbued with the necessity of proper education along these lines for dental students. I have in the past endeavored to connect this line of thought with our college work, but until the past year, so far as my knowledge extends, there has been nothing like a complete course in this work attempted by the colleges. The college with which I am connected gave a course in Physical Diagnosis last year. As one of the speakers has said, this is a subject that cannot well be taught by lectures alone, but must be demonstrated. Students have had opportunity to study these methods on living subjects, and we have found it anything but a dry subject. I believe I am correct in stating that greater interest was not manifested by the class in connection with any other work.

I most heartily endorse the broad scope that Dr. Rhein has given to the work. I did not speak of all the subjects looking to a higher preliminary education, because that would have made the paper too long. The omission was by no means because I have not given them much thought.

(To be continued.)

